# Systolic blood pressure and future stroke risk by asymptomatic brain lesions in a community MRI cohort: a retrospective study

**DOI:** 10.1038/s41440-026-02639-z

**Published:** 2026-04-22

**Authors:** Kenichi Iwasa, Naoki Omori, Shun Aritake, Yoshihito Aoki, Yukie Kanai, Atsushi Nagai

**Affiliations:** Department of Neurology, Faculty of Medicine, Shimane University, Izumo, Japan

**Keywords:** Asymptomatic brain lesions, Stroke, MRI, Implemental hypertension, Digital hypertension

## Abstract

Asymptomatic brain lesions (ABLs), including white matter hyperintensities (WMHs), silent brain infarcts (SBIs), and cerebral microbleeds (CMBs), are common MRI markers of cerebral small-vessel disease and predictors of future stroke. However, the optimal blood pressure (BP) target for primary prevention in individuals with ABLs remains unclear. We analyzed 2363 neurologically healthy adults (mean age 61.9 ± 10.9 years; 54% men) who underwent brain MRI screening (“Brain Dock”) and were followed for incident stroke over a mean of 8.9 years. ABLs were defined as the presence of SBI, WMH (Fazekas grade ≥ 2), or CMBs. The association between systolic BP (SBP) and stroke risk was evaluated using Cox proportional hazards models with restricted cubic splines, stratified by ABL status, and adjusted for age, sex, HbA1c, LDL-C, and antihypertensive use. The interaction between SBP and the ABL status was not significant. Stroke risk increased progressively with increasing SBP in both groups. In the ABL-positive group, the risk appeared to increase at comparatively lower SBP levels; however, this observation was descriptive and should not be interpreted as indicating a specific threshold. Due to limited stroke events and wide confidence intervals, particularly at higher SBP levels, these spline-based patterns should be considered exploratory rather than definitive. However, confirmation in larger prospective cohorts and interventional studies is needed before MRI-defined cerebrovascular markers can inform clinical blood pressure strategies.

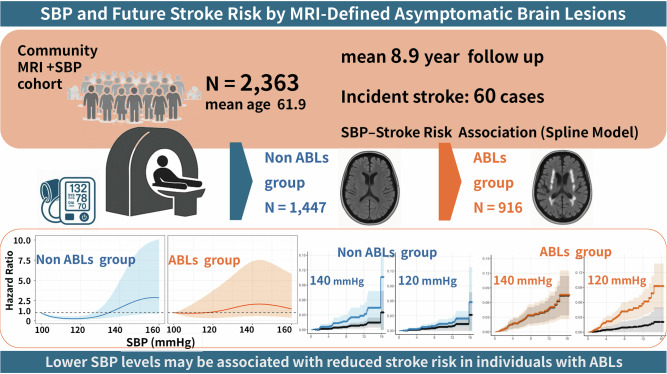

## Introduction

White matter hyperintensity (WMH), silent brain infarction (SBI), and cerebral microbleeds (CMBs), which are manifestations of cerebral small-vessel disease, are recognized as predictors of future stroke and cognitive decline in healthy individuals [1–4]. Collectively referred to as asymptomatic brain lesion (ABL), these findings are clinically silent but reflect chronic small-vessel injury and are associated with impaired cerebral autoregulation, microvascular fragility, and increased vulnerability to both ischemic and hemorrhagic stroke [5–7]. Furthermore, hypertension is a major risk factor for the development of ABLs [8–10].

Despite their prognostic significance, the current international hypertension guidelines provide no clear consensus on optimal blood pressure (BP) management among individuals with ABLs. Recent European guidelines emphasize the clinical importance of covert cerebral small-vessel disease but acknowledge that evidence is still insufficient to determine individualized BP thresholds based on MRI findings [11].

Recent large randomized controlled trials, including SPRINT and STEP, demonstrated that intensive BP lowering targeting systolic BP (SBP) ≤ 120 mmHg conferred significant cardiovascular benefits in high-risk populations [12, 13]. Consistent with these findings, recent Japanese studies have also demonstrated similar associations between elevated blood pressure and cerebrovascular outcomes. Both real-world data and a longitudinal community MRI cohort showed that higher SBP levels were continuously related to increased risks of stroke and progression of cerebral small-vessel disease [14, 15]. However, neuroimaging markers, such as ABLs, were not incorporated into their baseline risk stratification models. In the SPRINT-MIND MRI sub-study, intensive BP control below 120 mmHg was associated with significantly less progression of WMHs; however, the analysis was limited to 670 participants and did not assess incident stroke events within that cohort [16, 17]. The main analyses of both SPRINT and STEP confirmed reductions in overall cardiovascular risk, including stroke, under intensive BP-lowering, but neither trial evaluated baseline MRI characteristics or the modifying effects of silent cerebrovascular lesions.

In Japan, a nationwide preventive health screening program known as the “Brain Dock” has been established, which includes brain MRI for individuals without a prior history of neurological disease, primarily aimed at primary prevention of cerebrovascular disorders. Since the 1990s, the Brain Dock program has been implemented as part of Japan’s national preventive health initiatives, offering periodic MRI screening to local residents who voluntarily participate and pay out-of-pocket. Routine MRI screening for primary prevention is uncommon and is not widely recommended internationally, making this a unique system developed in Japan, where MRI is broadly accessible.

However, a lack of robust evidence supporting the utility of such screening programs has hindered the establishment of a global consensus on their clinical value. Although abnormalities such as cerebral aneurysms, brain tumors, or severe vascular stenosis detected during Brain Docking screening can prompt immediate referral for medical evaluation, the more commonly observed ABLs, which are well-established cerebrovascular risk markers, lack clear clinical intervention thresholds or management targets, thereby limiting the effectiveness of the program as a preventive health strategy.

Previous work from our group demonstrated that MRI-detected asymptomatic brain lesions could stratify future vascular risk in a community cohort, supporting the rationale for integrating MRI-based markers into stroke risk prediction frameworks [18]. Building upon this concept, the present study further investigates how systolic blood pressure interacts with these MRI-defined lesions to influence subsequent stroke risk.

This retrospective cohort study aimed to evaluate the association between BP and future stroke risk, based on the presence or absence of ABLs, in a large population of independent adults undergoing preventive brain MRI screening. Based on previous reports, we hypothesized that individuals with ABLs would be more susceptible to stroke, even at lower SBP levels, and that these imaging markers could help refine strategies for primary stroke prevention. To test this hypothesis, we utilized the Brain Dock database to analyze baseline SBP measurements, MRI findings, and incident stroke events during a longitudinal follow-up.

Point of view

- Clinical relevance
  MRI-detected asymptomatic brain lesions may identify individuals with increased cerebrovascular vulnerability to elevated systolic blood pressure.
- Future direction
  Large prospective studies are needed to determine whether neuroimaging markers can inform individualized blood pressure targets for primary stroke prevention.
- Consideration for the Asian population
  Given the high prevalence of cerebral small-vessel disease and the substantial burden of stroke in Asia, imaging-based risk stratification may help refine hypertension management strategies in Asian populations.

## Methods

### Study design and population

This retrospective cohort study adhered to the Strengthening the Reporting of Observational Studies in Epidemiology (STROBE) guidelines and was approved by the Ethics Committee of the Faculty of Medicine, Shimane University (Approval No. 2225) [19]. Written informed consent was obtained from all participants.

A total of 3853 neurologically healthy adults who underwent “Brain Dock” screening at the Shimane Institute of Health Science between July 2004 and November 2019 were considered eligible for inclusion. These participants voluntarily underwent screening primarily for the primary prevention of cerebrovascular diseases, paid for the examination out of their pocket, and received no financial compensation for their participation.

In addition to brain MRI, the Brain Dock program included regular fasting blood examinations and structured nurse-led interviews. The interviews collected self-reported information on medical history, medication use, and lifestyle factors such as smoking and alcohol consumption. Baseline data obtained at the time of screening were used to define cardiovascular risk factors for the present study. Incident stroke events were assessed annually using follow-up surveys conducted via telephone interviews or mailed questionnaires. Participants or their cohabiting family members were contacted to verify the occurrence and timing of any stroke, and medical documentation was obtained whenever possible. The Brain Dock program is a self-paid community-based health-screening service in Japan, and participants are generally considered relatively health-conscious individuals who voluntarily undergo screening examinations. Additionally, some municipalities provide partial financial support for brain-docking screening, particularly for older adults or specific target populations.

**Fig. 1:**
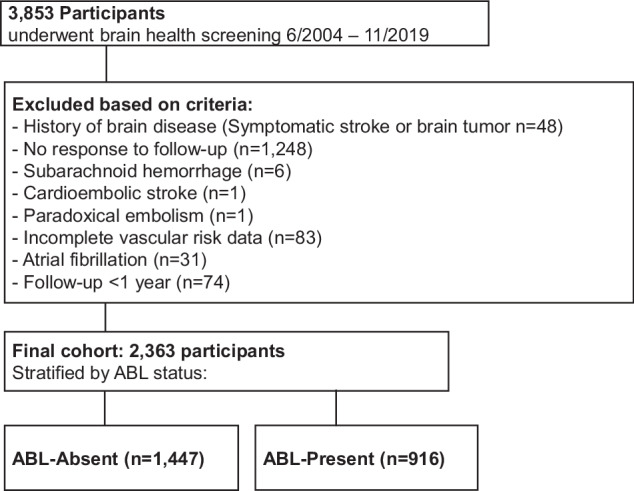
Study flow diagram. Flowchart showing participant selection from the initial 3853 adults who underwent Brain Dock MRI screening. Exclusion criteria included prior stroke or neurological disease, incomplete data, or follow-up < 1 year. In total, 2363 participants were included in the final analysis

Individuals with a history of symptomatic stroke, subarachnoid hemorrhage, traumatic brain injury, brain tumor, atrial fibrillation, cardioembolic or paradoxical embolism, incomplete vascular data, or a follow-up duration less than one year without stroke occurrence were excluded. Finally, 2363 participants were included in the final analysis (Fig. 1).

### Outcome ascertainment

The primary endpoint was occurrence of symptomatic stroke during the follow-up period. Stroke events were identified annually through telephone or mailed surveys based on self-reported or family-reported information.

### MRI acquisition and interpretation

MRI was performed using a 3.0-T Philips Ingenia system (Philips Healthcare). The imaging protocol included axial T1-weighted, T2-weighted, T2*-weighted, and fluid-attenuated inversion recovery (FLAIR) sequences with a slice thickness of 7 mm. Coronal and sagittal T1-weighted images were acquired.

For voxel-based morphometry (VBM) analysis, a three-dimensional T1-weighted image was acquired using an MP-RAGE sequence with the following parameters: repetition time (TR) = 6.8 ms, echo time (TE) = 3.1 ms, flip angle = 9°, number of sagittal slices = 170, in-plane resolution = 256 × 256, field of view = 256 × 256 mm, and voxel size = 1.0 × 1.0 × 1.2 mm.

Lesion classification was primarily based on the 2019 Brain Dock Guidelines from the Japan Brain Dock Society, complemented by established international criteria, such as the Fazekas scale for white matter lesions and the Brain Observer Microbleeds Scale (BOMBS) for CMBs, to ensure robust and reproducible definitions. All images were independently assessed by a board-certified neuroradiologist blinded to the participants’ clinical information.

### Definition of ABL

ABLs were defined according to the American Heart Association/American Stroke Association (AHA/ASA) Scientific Statement on the Prevention of Stroke in Patients with Silent Cerebrovascular Disease [20] and the STRIVE (Standards for Reporting Vascular Changes on Neuroimaging) [21] criteria. Participants were classified as ABL positive if at least one of the following imaging findings was present: SBI, WMH of presumed vascular origin, or CMBs.

Additionally, to explore the potential heterogeneity within the ABL-positive group, lesion-specific analyses were conducted and are presented descriptively in the Supplementary Materials.

#### SBI

Focal lesions consistent with previous ischemic infarctions were identified using MRI in the absence of a corresponding clinical history of stroke symptoms. Lesions typically appeared as areas of hyperintensity on T2/FLAIR and hypointensity on T1-weighted images, with or without central cavitation, and may involve either subcortical or cortical regions. SBI was differentiated from perivascular spaces by morphology, signal characteristics, and size (generally ≥3 mm). This definition encompasses both lacunar and non-lacunar silent infarcts, which is consistent with the AHA/ASA statement.

#### WMH

These were defined as areas of increased signal intensity on T2-weighted or FLAIR MRI consistent with ischemic small-vessel disease and were rated using the Fazekas scale. In accordance with prior AHA/ASA and STRIVE recommendations, deep/subcortical WMH grade ≥2 or periventricular hyperintensity grade ≥2 was regarded as abnormal and considered to reflect clinically relevant ischemic small-vessel disease.

#### CMBs

These were defined as small (2–10 mm), round, homogeneous hypointense lesions on T2-weighted gradient echo or susceptibility-weighted imaging (SWI). consistent with hemosiderin deposition from a prior microhemorrhage and clearly differentiated from calcifications or vascular flow voids.

The above operational definition aligns with the AHA/ASA Scientific Statement, which recognizes SBIs, WMHs of presumed vascular origin, and CMBs as the three cardinal manifestations of silent cerebrovascular disease that predict future symptomatic stroke and cognitive decline. Accordingly, participants were classified as ABL-positive if they had any of the following: SBI, periventricular hyperintensity (PVH) ≥ Grade 2, deep/subcortical white matter hyperintensity (DSWMH) ≥ Grade 2, or CMBs.

### Covariates and clinical definitions

As only 60 incident stroke events occurred, the number of covariates was restricted to six.

The covariates included systolic blood pressure (SBP), age, sex, use of antihypertensive medication (yes/no), hemoglobin A1c (HbA1c, %), and low-density lipoprotein cholesterol (LDL-C, mg/dL). Although additional clinical history and laboratory variables were available, we limited the adjustment variables to these factors to avoid model overfitting, given the small number of outcome events.

#### Clinical variables

SBP was measured once at the time of MRI screening using a standard automated digital sphygmomanometer after the participants rested in a seated position for at least 5 min. No repeated or ambulatory blood pressure monitoring (ABPM) was performed.

### Statistical analysis

#### Primary analysis

Model diagnostics were performed using Schoenfeld residuals and time-dependent interaction terms for SBP and ABL status to test the proportional hazard assumption. No substantial violations requiring time-dependent effects were observed, and the global test indicated an adequate model fit (*p* = 0.34). The corresponding Schoenfeld residual plots for each covariate are shown in Supplementary Fig. S1.

The association between baseline SBP and incident stroke was evaluated using Cox proportional hazards models, with time-to-event as the outcome. SBP was modeled as a continuous variable using a restricted cubic spline (RCS) with three degrees of freedom to flexibly capture nonlinear associations. The models were stratified by ABL status (present or absent), and the reference value for SBP was set at 100 mmHg.

The hazard ratio (HR) for stroke at a given SBP (*x*) was estimated as follows:

$${{{\rm{HR}}}}({{{\rm{x}}}})=\exp \;[{{{{\rm{\beta }}}}}^{{{\top }}}\{{{{\rm{f}}}}({{{\rm{x}}}})-{{{\rm{f}}}}(100)\}],$$

where f(x) denotes the spline basis expansion of SBP and β represents the estimated coefficients.

The primary hypothesis was to determine whether there is a statistical interaction between SBP (modeled as a continuous variable using splines) and ABL status. The Wald chi-square test was applied to the pooled estimates obtained after multiple imputations. The multivariate model was adjusted for prespecified confounders: age, sex, HbA1c, LDL-C, and antihypertensive medication use. Serum HbA1c and low-density lipoprotein cholesterol (LDL-C) levels were measured under fasting conditions using standard enzymatic methods in certified laboratories.

#### Spline model specification

Restricted cubic spline models were fitted with three degrees of freedom using two internal knots placed at the 33rd and 66th percentiles of the SBP distribution. This approach allowed for a smooth estimation of the continuous SBP effect while avoiding abrupt changes in the HRs between adjacent values.

A reference SBP of 100 mmHg was selected to enhance the visual interpretability of risk transitions.

#### Handling of missing data

Missing covariate data were imputed 20 times, using multiple imputations with chained equations (MICE). Outcome variables (follow-up time and stroke occurrence) were not imputed.

The imputation model included all the covariates, exposure variables, and relevant auxiliary clinical data. Rubin’s rule was applied to combine parameter estimates across the imputed datasets. A full list of variables with missing data counts and proportions is shown in Supplementary Table S1.

#### Sensitivity analysis

Sensitivity analyses the robustness of findings. First, participants with intracranial arterial stenosis on baseline magnetic resonance angiography were excluded. Second, participants who initiated antihypertensive therapy within one year of baseline screening were excluded. Finally, to evaluate potential selection bias from loss to follow-up, baseline characteristics were compared between participants with complete follow-up and those without follow-up responses using effect size–based methods.

To address potential model instability due to the limited number of stroke events, we conducted a sensitivity analysis incorporating the Disease Risk Score (DRS). The DRS was derived as a linear predictor from a Cox model including age, sex, HbA1c, LDL-C, and antihypertensive use, and was entered as a continuous covariate in the main spline-based SBP–ABL interaction model.

#### Internal validation

The internal validity of the Cox proportional hazards model was evaluated using bootstrap resampling with 1000 iterations. The optimism-corrected performance metrics, including the C-index, calibration slope, and explained variance (*R*²), were computed and compared with those obtained from a ridge-regularized model (penalty = 1).

#### Subgroup analyses

Subgroup analyses were additionally performed for the stroke subtypes (ischemic stroke and intracerebral hemorrhage). The SBP–ABL interaction was examined separately for each subtype using the same modeling strategy as in the primary analysis, and three-dimensional spline curves were generated to visualize the subtype-specific risk patterns.

Additional subgroup analyses were conducted for each ABL component (PVH grade ≥ 2, DSWMH grade ≥ 2, SBI, and CMBs). The association between SBP and stroke risk was examined in each subgroup using the same spline-based approach, and three-dimensional plots were created to visualize the corresponding five-year absolute risk surfaces.

#### Cumulative hazard analysis by representative SBP categories

Cumulative hazards throughout the follow-up period were evaluated for representative SBP categories of 120, 130, and 140 mmHg, stratified by ABL status. Cox proportional hazards models were used to estimate and plot cumulative hazards for each SBP category.

All statistical analyses were performed using the R software (RStudio Ver. 2025.09.1 Build 401, R Foundation for Statistical Computing). Statistical significance was defined as a two-sided *p*-value of < 0.05.

## Results

### Study population

A total of 2363 participants (mean age, 61.9 ± 10.9 years; 54% men) were included in the final analysis. Based on baseline MRI findings, 916 participants (38.8%) were classified as ABL-positive, defined by the presence of SBIs, WMH of Fazekas grade ≥2, or CMBs. Baseline characteristics stratified by ABL status are summarized in Table 1.

**Table 1:** Baseline characteristics of participants stratified by asymptomatic brain lesion (ABL) status

Variable	ABL-Absent (*N* = 1447)	ABL-Present (*N* = 916)	*p*-value
**Continuous variables**			
Age, years	57.8 ± 10.3	68.5 ± 8.2	<0.001
SBP, mmHg	126 ± 17.3	132 ± 18.0	<0.001
DBP, mmHg	73.1 ± 11.3	74.0 ± 10.9	0.04
BMI, kg/m²	23.1 ± 3.2	22.9 ± 2.9	0.15
HbA1c, %	5.43 ± 0.60	5.58 ± 0.73	<0.001
TG, mg/dL	117.5 ± 77.4	112.5 ± 63.3	0.12
HDL-C, mg/dL	62.8 ± 16.0	62.4 ± 16.7	0.48
LDL-C, mg/dL	123.2 ± 29.8	120.8 ± 29.5	0.09
Creatinine, mg/dL	0.74 ± 0.16	0.75 ± 0.23	0.24
Categorical variables			
Male	802 (55.4%)	462 (50.4%)	0.018
Hypertension	752 (52.0%)	623 (68.0%)	<0.001
Antihypertensive use	274 (18.9%)	351 (38.3%)	<0.001
Antiplatelet use	69 (4.8%)	86 (9.4%)	<0.001
Diabetes mellitus	133 (9.2%)	150 (16.4%)	<0.001
Dyslipidemia	871 (60.2%)	553 (60.4%)	0.931
Ever smoker	389(26.9%)	216(23.6%)	0.073
MRI findings			
PVH ≥ 2	0 (0.0%)	521 (56.9%)	<0.001
DSWMH ≥ 2	0 (0.0%)	682 (74.5%)	<0.001
Silent brain infarct	0 (0.0%)	135 (14.7%)	<0.001
Microbleeds	0 (0.0%)	158 (17.2%)	<0.001
Outcomes			
Follow-up duration, year	9.05 ± 4.36	8.55 ± 4.65	0.009
Stroke	22 (1.5%)	38 (4.1%)	<0.001
Ischemic stroke	18 (1.2%)	29 (3.2%)	0.001
Hemorrhagic stroke	4 (0.3%)	9 (1.0%)	0.041

Values are presented as mean ± SD for continuous variables and n (%) for categorical variables

*p*-values derived from independent *t*-tests for continuous variables and *χ*^2^ tests for categorical variables. Magnetic resonance imaging (MRI) findings were assessed using the Fazekas scale and the standard definitions of silent brain infarcts and microbleeds

Outcomes represent cumulative incidence during the observation period (mean follow-up 8.9 years). Stroke subtypes were confirmed by clinical records or participant/family reports validated by medical documentation

Effect-size-based comparisons showed negligible baseline differences between participants with and without complete follow-up. For continuous variables, all Cohen’s *d* values were near zero with confidence intervals spanning zero; for categorical variables, all Cramér’s *V* values were ≤ 0.02 (Supplementary Tables S2 ans S3).

Compared with ABL-negative participants, those with ABLs were significantly older and had higher systolic BP. They were also more likely to be male, have diabetes mellitus, and use antihypertensive and antiplatelet medications. While HbA1c levels differed significantly between the groups, LDL cholesterol and serum creatinine levels did not. The proportion of missing data was minimal across all variables, with the highest rate observed for antihypertensive medication use (3.3%). The details of the missing data counts and proportions for each variable are presented in Supplementary Table S1. Missing values were handled by multiple imputations using chained equations.

#### Incident stroke events

During a mean follow-up of 8.9 years (SD 4.5 years), 60 stroke events (2.5%) occurred. Of these, 47 (78.3%) had ischemic stroke, and 13 (21.7%) had intracerebral hemorrhage.

### Association of SBP with stroke risk by ABL status

**Fig. 2:**
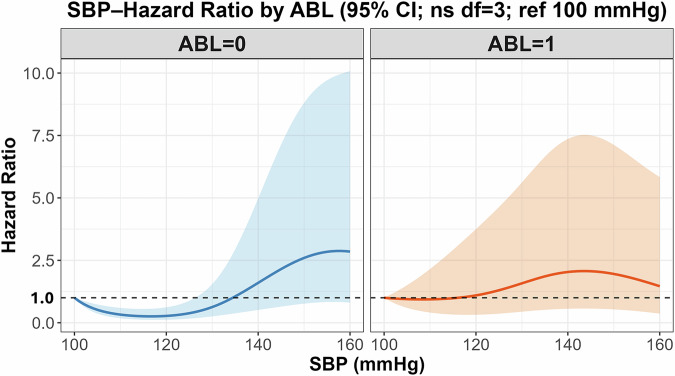
Adjusted hazard ratio curves for stroke according to systolic blood pressure (SBP), stratified by asymptomatic brain lesion (ABL) status. Restricted cubic spline models with 3 degrees of freedom were adjusted for age, sex, HbA1c level, LDL-C level, and antihypertensive use. Solid lines represent hazard ratio estimates (blue = ABL-negative; red = ABL-positive) and shaded areas indicate 95% confidence intervals. Reference value: SBP = 100 mmHg. Numerical HR estimates corresponding to these curves are provided in Supplementary Table S4

Using 100 mmHg as the reference value, restricted cubic spline models with three degrees of freedom were constructed to depict the association between SBP and stroke hazard (Fig. 2). The statistical interaction between SBP and ABL status was not significant (*p* = 0.059). Both groups showed a similar pattern of higher estimated stroke risk with higher SBP. While the curves suggest risk elevation may occur at lower SBP ranges in ABL-positive participants, apparent inflection points should not be interpreted as definitive thresholds, given limited events and wide confidence intervals. The numerical hazard ratio estimates across the full SBP range corresponding to the spline curves in Fig. 2 are listed in Supplementary Table S4.

Internal validity assessed by bootstrap resampling (1000 iterations) yielded an optimism-corrected C-index of 0.707, indicating moderate discriminative ability, and a calibration slope of 0.70. Ridge regularization slightly improved the calibration stability without compromising discrimination. The explained variance (*R*²) was 0.03, indicating a limited number of events. In the sensitivity analysis incorporating the disease risk score (DRS), the direction of the SBP–ABL interaction remained consistent with the primary analysis. The Wald test for the interaction term yielded *χ*^2^ = 7.19 (df = 3, *p* = 0.066), not reaching statistical significance. The confidence intervals of the spline curves widened at higher SBP levels, reflecting the limited number of stroke events. Therefore, the results should be interpreted with caution, particularly in extreme SBP ranges.

### Five-year absolute risk estimation

**Fig. 3:**
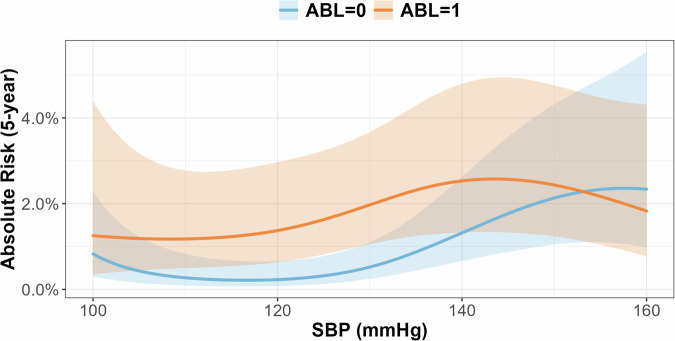
Five-year absolute risk of stroke across systolic blood pressure (SBP) levels by asymptomatic brain lesion (ABL) status. Five-year absolute stroke risk estimated from multivariable Cox models with restricted cubic splines by systolic blood pressure (SBP) and MRI-defined asymptomatic brain lesion (ABL) status. ABL-positive participants showed higher stroke risk across SBP range, with greater separation at some SBP levels. These estimates are exploratory and should not be interpreted as evidence of a specific SBP threshold

Three-dimensional plots illustrating the five-year absolute risk of stroke according to SBP and ABL status are shown in Fig. 3. Overall, the participants with ABLs exhibited a higher estimated stroke risk across the SBP range; however, the uncertainty remained substantial.

### Subgroup analyses

#### Risk comparison by stroke subtype

##### Ischemic stroke

In the subgroup analysis, the outcome was stratified by stroke subtype (ischemic stroke and intracerebral hemorrhage), and the SBP–ABL interaction was examined using the same modeling strategy as in the primary analysis.

**Fig. 4:**
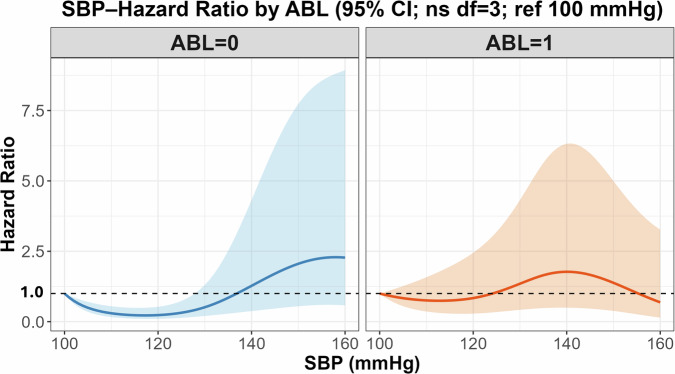
Adjusted hazard ratio curves for ischemic stroke by systolic blood pressure (SBP) and asymptomatic brain lesion (ABL) status. Restricted cubic spline models for the ischemic stroke subtype showed no significant SBP–ABL interaction (*p* = 0.0503). Given the limited number of events and wide confidence intervals, these curves are presented descriptively and should be regarded as hypothesis-generating

The SBP-ABL interaction did not reach significance in the analysis of the 47 ischemic stroke cases (*p* = 0.0503). Given the limited number of events, the confidence intervals were wide, and subtype-specific spline curves were presented descriptively to illustrate possible patterns (Fig. 4).

##### Hemorrhagic stroke

For intracerebral hemorrhage, the interaction test resulted in a *p*-value of 0.70, indicating no significant association. The spline curve could not be accurately described because of the small number of hemorrhagic events.

##### Additional subgroup analyses by lesion components

Further subgroup analyses were conducted for individual lesion components, including SBI, PVH ≥ 2, DSWMH ≥ 2, and CMBs.

Although the shapes of the spline-based risk curves varied across lesion types, they all consistently showed a higher stroke risk among lesion-positive participants than among lesion-negative participants. Detailed results and corresponding curves are provided in Supplementary Fig. S2. In lesion-specific analyses among ABL-positive participants (Supplementary Fig. S2), SBP–stroke risk spline curves differed across lesion subtypes. The spline patterns for white matter hyperintensities, including deep/subcortical and periventricular lesions, most closely resembled the overall curve in the composite ABL-positive group, while curves for silent brain infarctions and cerebral microbleeds were less stable, with wider confidence intervals.

### Cumulative hazards by representative SBP categories

**Fig. 5:**
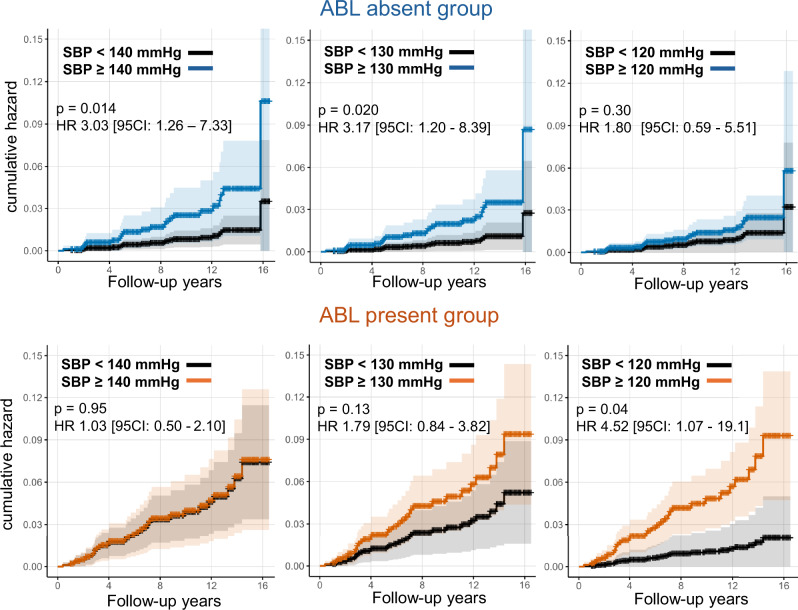
Cumulative hazard curves for stroke according to representative SBP categories (120, 130, and 140 mmHg), stratified by ABL status. Cumulative hazards estimated over the entire follow-up period. Blue lines denote ABL-negative and red lines ABL-positive participants. Visual differences were observed across representative SBP categories; however, these comparisons are exploratory

The cumulative hazards of stroke across representative SBP categories (120, 130, and 140 mmHg) stratified by ABL status are shown in Fig. 5. Among ABL-negative participants, the risk differences between SBP 120 mmHg and higher categories (130–140 mmHg) were minimal, whereas in ABL-positive individuals, a visual risk separation was observed across representative SBP categories. This comparison was exploratory, without adjustment for multiple comparisons. These plots were intended for visualization and should not be used to define clinical SBP targets or cut-offs.

### Sensitivity analysis

After excluding participants with intracranial arterial stenosis (*n* = 2317), the spline curves showed patterns similar to the primary analysis, preserving the differential SBP–stroke risk relationship by ABL status (Supplementary Fig. S3). After excluding participants who initiated antihypertensive therapy within one year of baseline screening (*n* = 2343), the SBP–stroke spline curves remained consistent with main findings (Supplementary Fig. S4). In a sensitivity analysis adjusted for DRS as a covariate, the shapes of the SBP–stroke risk spline curves by ABL status remained largely unchanged compared with the primary analysis (Supplementary Fig. S5).

## Discussion

This study examined the association between SBP and future stroke risk, based on the presence of ABLs, using a large MRI-based cohort derived from a community health screening program. Although the interaction between SBP and ABL status did not reach statistical significance, a higher SBP tended to be associated with an increased risk of stroke, regardless of the ABL status. In individuals with ABLs, risk increased at lower SBP levels; however, this observation is descriptive and should not indicate a specific threshold.

A key limitation is the small number of incident stroke events, which limits statistical power and increases uncertainty. The confidence intervals widened at higher SBP levels, reflecting limited stroke events; therefore, observed patterns should emphasize overall trends rather than point estimates.

Our findings are consistent with the possibility that ABLs may serve as potential imaging markers for personalized approaches for hypertension management. Given the observed trends, ABL-positive individuals may have reduced cerebrovascular tolerance to elevated BP [18], and the hypothesis that lower SBP may be associated with a lower stroke risk in this subgroup warrants careful consideration. These inferences should be regarded as exploratory and require confirmation in future prospective studies. This interpretation is cautiously consistent with findings from post hoc analyses of the RESPECT trial, in which intensive BP control was associated with lower recurrence of both ischemic and hemorrhagic stroke among Japanese patients with prior cerebrovascular disease [22].

Current hypertension guidelines, including the 2025 AHA/ACC recommendation [23], generally advocate a target SBP of <130 mmHg for most adults, based on office or home measurements. However, these guidelines do not provide specific recommendations for individuals with asymptomatic intracranial lesions detected by MRI. In this context, the presence of ABLs, although clinically silent, may reflect underlying cerebral small-vessel vulnerability, potentially modifying tolerance to higher BP.

Therefore, MRI-based detection of ABLs could serve as a useful exploratory tool for identifying subgroups that warrant further investigation of BP control intensity. Nevertheless, this hypothesis requires validation in prospective and interventional studies before it can be incorporated into clinical practice. This approach is conceptually consistent with the framework of precision prevention in which risk stratification using imaging or biomarker data enables targeted interventions. This paradigm has been successfully applied to other chronic conditions, including diabetes and chronic kidney disease [24, 25], and may provide a framework for future investigations on hypertension management when integrated with MRI-based cerebrovascular markers.

The SBP–stroke risk spline curves remained largely unchanged across sensitivity analyses, including exclusion of participants with intracranial arterial stenosis, exclusion of those initiating antihypertensive therapy within one year after baseline, and additional adjustment for DRS, supporting the robustness of observed patterns.

Although asymptomatic brain lesions represent a heterogeneous group, lesion-specific analyses provide insights into the composite findings. As shown in Supplementary Fig. S2, the non-linear SBP-stroke risk pattern in the ABL-positive group was most closely reflected by white matter hyperintensities, the most prevalent lesion type in this cohort. This suggests white matter lesions largely contribute to the composite ABL-associated risk profile. Importantly, this interpretation is descriptive and hypothesis-generating, without attributing causal or hierarchical importance to individual lesion subtypes.

### Strengths and limitations

The strengths of this study include:

1. Using a large, long-term, community-based cohort focused on primary prevention.
2. Standardized MRI-based assessment of brain lesions
3. Applying spline-based modeling to examine continuous SBP risk associations across a wide range of values.

This approach allowed smooth visualization and comparison of SBP risk curves according to the ABL status.

However, some limitations of the study should be acknowledged. First, stroke events were identified from self- or family-reported information, which may have introduced an information bias. Second, BP was measured only once at baseline; therefore, the possible effects of diurnal variations or white-coat hypertension cannot be excluded. Additionally, masked hypertension could not be assessed, and these factors may have attenuated or distorted the observed SBP–stroke association. Third, changes in antihypertensive therapy or lifestyle factors during the follow-up were not fully captured. Screening results feedback may have prompted medical consultation and treatment changes, particularly among participants with ABLs or elevated BP, which could have biased associations toward the null. Fourth, as an observational design, causal inference cannot be established. Finally, follow-up MRI data were unavailable, precluding the assessment of the temporal relationship between ABL progression and stroke occurrence. Fifth, because Brain Dock screening is primarily self-paid, participants may represent a health-conscious population. However, in some municipalities, partial public support is provided to selected groups, which may broaden participant representation. Therefore, the generalizability of the present findings to the broader general population should still be interpreted with caution. Although we adjusted for glycemic status (HbA1c), residual confounding by diabetes-related factors like disease duration, complications, and treatment changes cannot be excluded. Additionally, data on ischemic heart disease and peripheral arterial disease incidences were not collected for this cohort. These vascular comorbidities share atherosclerotic risk factors with ischemic stroke and may act as confounders in stroke risk assessment and influence blood pressure management strategies. While major cardiovascular risk factors were adjusted for in multivariate models, residual confounding factors related to systemic atherosclerotic burden cannot be excluded. These limitations should be considered when interpreting our findings that reflect tendencies rather than definitive causal relationships.

Effect-size comparisons showed negligible baseline differences between participants with complete follow-up and those without follow-up responses, suggesting selection bias due to loss to follow-up unlikely influenced results.

In conclusion, our analyses revealed trends suggesting that ABLs may be potential imaging markers for refining individualized BP management strategies for primary stroke prevention. Although the interaction between SBP and ABL status was not significant, stroke risk increased with higher SBP across participants, with hazards rising at lower SBP levels in those with ABLs. These observations should be interpreted as exploratory, emphasizing confidence intervals and trends rather than point estimates or specific SBP cutoffs. Confirmation in larger cohorts and interventional studies is essential before proposing MRI-based BP targets and recommendations.

### Perspective of asia

Hypertension remains the leading modifiable risk factor for stroke worldwide and plays a particularly prominent role in Asian populations. Large international epidemiological studies have consistently shown that elevated blood pressure accounts for the largest proportion of attributable stroke risk globally [26]. In addition, the burden of cerebrovascular disease is substantially higher in Asia than in many Western regions, with East Asian countries reporting some of the highest stroke incidence and mortality rates worldwide [27].

Importantly, the epidemiological characteristics of stroke differ between Asian and Western populations. Previous studies have documented a higher proportion of intracerebral hemorrhage and a greater prevalence of cerebral small-vessel disease in Asian populations, both of which are strongly associated with chronic hypertension and vascular fragility [28]. These regional differences suggest that cerebrovascular susceptibility to elevated blood pressure may vary across populations and may influence optimal strategies for hypertension management.

In this context, neuroimaging markers of subclinical cerebrovascular injury may provide additional insight into risk stratification for hypertension management. Asymptomatic brain lesions (ABLs), including white matter hyperintensities, silent brain infarctions, and cerebral microbleeds, represent key imaging manifestations of cerebral small-vessel disease and are increasingly recognized as predictors of future cerebrovascular events.

Japan provides a unique clinical framework through the community-based MRI screening program known as “Brain Dock,” which enables the detection of subclinical cerebrovascular abnormalities in neurologically healthy individuals. Leveraging this resource, the present study examined the association between systolic blood pressure and future stroke risk in a large MRI-based community cohort.

Although the statistical interaction between SBP and ABL status was not significant, the observed risk patterns suggest that individuals with MRI-detected cerebrovascular lesions may have increased vulnerability to elevated blood pressure. Given the substantial burden of stroke in Asia and the expanding availability of neuroimaging across several Asian countries, incorporating imaging markers of cerebral small-vessel disease into cardiovascular risk assessment may contribute to the development of more individualized strategies for stroke prevention in Asian populations.

## Supplementary information

Supplementary Table S1

Supplementary Table S2

Supplementary Table S4

Supplementary Figure S1

Supplementary Figure S2

Supplementary Figure S3

Supplementary Figure S4

Supplementary Figure S5
