# Supplementary material for: Systolic blood pressure and future stroke risk by asymptomatic brain lesions in a community MRI cohort: a retrospective study
Source: Hypertens Res. 2026 Apr 22;49(6):1866–77. doi: 10.1038/s41440-026-02639-z (PMC13236583; doi:10.1038/s41440-026-02639-z)
Supplement: Supplementary file 1 — Supplementary Table S1 [file 41440_2026_2639_MOESM1_ESM.docx]

**Supplementary Table S1. Missing data counts and proportions for all variables included in the analysis**

| **Variable** | **Missing_n** | **Missing_rate** |
| --- | --- | --- |
| Use of antihypertensive medication | 79 | 0.0334 |
| Ever smoker (yes/no) | 10 | 0.0042 |
| Hemoglobin A1c (%) | 3 | 0.0013 |
| Body mass index (kg/m²) | 1 | 0.0004 |
| Serum creatinine (mg/dL) | 1 | 0.0004 |
| Age (years) | 0 | 0 |
| Sex (male/female) | 0 | 0 |
| Systolic blood pressure (mmHg) | 0 | 0 |
| Diastolic blood pressure (mmHg) | 0 | 0 |
| History of hypertension | 0 | 0 |
| History of diabetes mellitus | 0 | 0 |
| History of dyslipidemia | 0 | 0 |
| Low-density lipoprotein cholesterol (mg/dL) | 0 | 0 |
| High-density lipoprotein cholesterol (mg/dL) | 0 | 0 |
| Triglycerides (mg/dL) | 0 | 0 |
| Use of antiplatelet agents | 0 | 0 |
| Silent brain infarcts (present/absent) | 0 | 0 |
| Periventricular white matter hyperintensities | 0 | 0 |
| Deep/subcortical white matter hyperintensities | 0 | 0 |
| Cerebral microbleeds (present/absent) | 0 | 0 |
| Incident stroke (any type) | 0 | 0 |
| Ischemic stroke | 0 | 0 |
| Hemorrhagic stroke | 0 | 0 |

Missing data counts and proportions for all variables included in the multiple imputation and main analyses. The overall amount of missing data was minimal (<5% for all the variables). Only *antihypertensive use* (3.3%) and *eversmoker status* (0.4%) showed minor missingness, which was addressed by multiple imputations using chained equations (m = 20). No missing data were present for outcomes or major exposure variables (SBP, ABL, and stroke).
