# Supplementary material for: Systolic blood pressure and future stroke risk by asymptomatic brain lesions in a community MRI cohort: a retrospective study
Source: Hypertens Res. 2026 Apr 22;49(6):1866–77. doi: 10.1038/s41440-026-02639-z (PMC13236583; doi:10.1038/s41440-026-02639-z)
Supplement: Supplementary file 2 — Supplementary Table S2 [file 41440_2026_2639_MOESM2_ESM.docx]

**Supplementary Table S2. Baseline continuous characteristics by follow-up response status (effect-size–based comparison)**

| **Variable** | **Effect size (Cohen’s d)** | **95% CI** |
| --- | --- | --- |
| Age (years) | −0.044 | −0.118 to 0.029 |
| BMI (kg/m²) | 0.015 | −0.059 to 0.088 |
| SBP (mmHg) | 0.046 | −0.027 to 0.120 |
| DBP (mmHg) | −0.041 | −0.114 to 0.033 |
| LDL-C (mg/dL) | 0.07 | −0.004 to 0.145 |
| HbA1c (%) | 0.009 | −0.065 to 0.083 |
| Creatinine (mg/dL) | 0.013 | −0.061 to 0.087 |

Effect sizes for categorical variables were assessed using Cramér’s V. Values close to zero indicate negligible differences between participants with and without follow-up responses.

**Supplementary table S3. Baseline categorical characteristics by follow-up response status (effect-size–based comparison)**

| **Variable** | **Effect size (Cramér’s V)** |
| --- | --- |
| Sex | 0.006 |
| Hypertension | 0.02 |
| Use Antihypertensive | 0.005 |
| Diabetes | 0.008 |
| ABL prevalence | 0.02 |

Effect sizes for categorical variables were assessed using Cramér’s V. Values close to zero indicate negligible differences between participants with and without follow-up responses.
