# Supplementary material for: Systolic blood pressure and future stroke risk by asymptomatic brain lesions in a community MRI cohort: a retrospective study
Source: Hypertens Res. 2026 Apr 22;49(6):1866–77. doi: 10.1038/s41440-026-02639-z (PMC13236583; doi:10.1038/s41440-026-02639-z)
Supplement: Supplementary file 3 — Supplementary Table S4 [file 41440_2026_2639_MOESM3_ESM.docx]

**Supplementary Table S4. Adjusted hazard ratios for incident stroke across continuous systolic blood pressure levels based on restricted cubic spline models stratified by ABL status**

|  | SBP (mmHg) | HR | 95%CI, lower | 95%CI upper |
| --- | --- | --- | --- | --- |
| ABL-absent group | 90 | 5.72 | 1.20 | 27.21 |
|  | 91 | 4.71 | 1.01 | 21.96 |
|  | 92 | 3.89 | 0.85 | 17.89 |
|  | 93 | 3.23 | 0.71 | 14.73 |
|  | 94 | 2.69 | 0.59 | 12.24 |
|  | 95 | 2.25 | 0.49 | 10.27 |
|  | 96 | 1.90 | 0.41 | 8.70 |
|  | 97 | 1.60 | 0.34 | 7.45 |
|  | 98 | 1.36 | 0.29 | 6.43 |
|  | 99 | 1.16 | 0.24 | 5.60 |
|  | 100 | 1.00 | 0.20 | 4.92 |
|  | 101 | 0.87 | 0.17 | 4.36 |
|  | 102 | 0.75 | 0.15 | 3.90 |
|  | 103 | 0.66 | 0.12 | 3.51 |
|  | 104 | 0.58 | 0.11 | 3.18 |
|  | 105 | 0.52 | 0.09 | 2.91 |
|  | 106 | 0.47 | 0.08 | 2.68 |
|  | 107 | 0.42 | 0.07 | 2.48 |
|  | 108 | 0.38 | 0.06 | 2.32 |
|  | 109 | 0.35 | 0.06 | 2.18 |
|  | 110 | 0.33 | 0.05 | 2.07 |
|  | 111 | 0.31 | 0.05 | 1.97 |
|  | 112 | 0.29 | 0.04 | 1.90 |
|  | 113 | 0.28 | 0.04 | 1.83 |
|  | 114 | 0.27 | 0.04 | 1.79 |
|  | 115 | 0.26 | 0.04 | 1.76 |
|  | 116 | 0.26 | 0.04 | 1.74 |
|  | 117 | 0.26 | 0.04 | 1.74 |
|  | 118 | 0.26 | 0.04 | 1.75 |
|  | 119 | 0.27 | 0.04 | 1.77 |
|  | 120 | 0.28 | 0.04 | 1.82 |
|  | 121 | 0.29 | 0.04 | 1.88 |
|  | 122 | 0.31 | 0.05 | 1.96 |
|  | 123 | 0.33 | 0.05 | 2.06 |
|  | 124 | 0.36 | 0.06 | 2.18 |
|  | 125 | 0.39 | 0.06 | 2.32 |
|  | 126 | 0.43 | 0.07 | 2.49 |
|  | 127 | 0.47 | 0.08 | 2.69 |
|  | 128 | 0.52 | 0.09 | 2.92 |
|  | 129 | 0.58 | 0.10 | 3.19 |
|  | 130 | 0.64 | 0.12 | 3.49 |
|  | 131 | 0.71 | 0.13 | 3.83 |
|  | 132 | 0.79 | 0.15 | 4.20 |
|  | 133 | 0.88 | 0.17 | 4.62 |
|  | 134 | 0.98 | 0.19 | 5.08 |
|  | 135 | 1.08 | 0.21 | 5.56 |
|  | 136 | 1.19 | 0.23 | 6.08 |
|  | 137 | 1.30 | 0.25 | 6.61 |
|  | 138 | 1.41 | 0.28 | 7.17 |
|  | 139 | 1.52 | 0.30 | 7.73 |
|  | 140 | 1.64 | 0.32 | 8.31 |
|  | 141 | 1.76 | 0.35 | 8.90 |
|  | 142 | 1.87 | 0.37 | 9.48 |
|  | 143 | 1.99 | 0.39 | 10.06 |
|  | 144 | 2.10 | 0.42 | 10.63 |
|  | 145 | 2.21 | 0.44 | 11.18 |
|  | 146 | 2.31 | 0.46 | 11.72 |
|  | 147 | 2.41 | 0.48 | 12.23 |
|  | 148 | 2.51 | 0.49 | 12.72 |
|  | 149 | 2.59 | 0.51 | 13.19 |
|  | 150 | 2.67 | 0.52 | 13.62 |
|  | 151 | 2.74 | 0.54 | 14.03 |
|  | 152 | 2.80 | 0.54 | 14.41 |
|  | 153 | 2.85 | 0.55 | 14.76 |
|  | 154 | 2.89 | 0.55 | 15.09 |
|  | 155 | 2.93 | 0.56 | 15.40 |
|  | 156 | 2.95 | 0.55 | 15.69 |
|  | 157 | 2.96 | 0.55 | 15.97 |
|  | 158 | 2.96 | 0.54 | 16.23 |
|  | 159 | 2.95 | 0.53 | 16.49 |
|  | 160 | 2.93 | 0.51 | 16.75 |
| ABL-present group | 90 | 1.22 | 0.03 | 43.36 |
|  | 91 | 1.19 | 0.04 | 37.84 |
|  | 92 | 1.17 | 0.04 | 33.13 |
|  | 93 | 1.14 | 0.04 | 29.09 |
|  | 94 | 1.11 | 0.05 | 25.64 |
|  | 95 | 1.09 | 0.05 | 22.68 |
|  | 96 | 1.07 | 0.06 | 20.13 |
|  | 97 | 1.05 | 0.06 | 17.95 |
|  | 98 | 1.03 | 0.07 | 16.07 |
|  | 99 | 1.01 | 0.07 | 14.45 |
|  | 100 | 1.00 | 0.08 | 13.06 |
|  | 101 | 0.99 | 0.08 | 11.85 |
|  | 102 | 0.98 | 0.09 | 10.81 |
|  | 103 | 0.97 | 0.09 | 9.91 |
|  | 104 | 0.96 | 0.10 | 9.13 |
|  | 105 | 0.95 | 0.11 | 8.46 |
|  | 106 | 0.95 | 0.11 | 7.88 |
|  | 107 | 0.94 | 0.12 | 7.38 |
|  | 108 | 0.94 | 0.13 | 6.95 |
|  | 109 | 0.94 | 0.13 | 6.58 |
|  | 110 | 0.94 | 0.14 | 6.26 |
|  | 111 | 0.95 | 0.15 | 6.00 |
|  | 112 | 0.96 | 0.16 | 5.77 |
|  | 113 | 0.97 | 0.17 | 5.59 |
|  | 114 | 0.98 | 0.18 | 5.44 |
|  | 115 | 0.99 | 0.18 | 5.32 |
|  | 116 | 1.01 | 0.19 | 5.24 |
|  | 117 | 1.03 | 0.20 | 5.19 |
|  | 118 | 1.05 | 0.21 | 5.17 |
|  | 119 | 1.08 | 0.22 | 5.18 |
|  | 120 | 1.11 | 0.24 | 5.23 |
|  | 121 | 1.14 | 0.25 | 5.30 |
|  | 122 | 1.18 | 0.26 | 5.42 |
|  | 123 | 1.22 | 0.27 | 5.57 |
|  | 124 | 1.27 | 0.28 | 5.75 |
|  | 125 | 1.32 | 0.29 | 5.96 |
|  | 126 | 1.37 | 0.30 | 6.21 |
|  | 127 | 1.43 | 0.31 | 6.50 |
|  | 128 | 1.48 | 0.32 | 6.81 |
|  | 129 | 1.54 | 0.33 | 7.16 |
|  | 130 | 1.60 | 0.34 | 7.54 |
|  | 131 | 1.66 | 0.35 | 7.94 |
|  | 132 | 1.72 | 0.36 | 8.36 |
|  | 133 | 1.78 | 0.36 | 8.79 |
|  | 134 | 1.84 | 0.37 | 9.22 |
|  | 135 | 1.89 | 0.37 | 9.64 |
|  | 136 | 1.94 | 0.37 | 10.04 |
|  | 137 | 1.98 | 0.38 | 10.41 |
|  | 138 | 2.02 | 0.38 | 10.74 |
|  | 139 | 2.05 | 0.38 | 11.03 |
|  | 140 | 2.07 | 0.38 | 11.29 |
|  | 141 | 2.09 | 0.38 | 11.49 |
|  | 142 | 2.11 | 0.38 | 11.65 |
|  | 143 | 2.11 | 0.38 | 11.76 |
|  | 144 | 2.12 | 0.38 | 11.83 |
|  | 145 | 2.11 | 0.38 | 11.84 |
|  | 146 | 2.10 | 0.37 | 11.81 |
|  | 147 | 2.08 | 0.37 | 11.74 |
|  | 148 | 2.06 | 0.37 | 11.63 |
|  | 149 | 2.03 | 0.36 | 11.48 |
|  | 150 | 2.00 | 0.36 | 11.30 |
|  | 151 | 1.97 | 0.35 | 11.09 |
|  | 152 | 1.93 | 0.34 | 10.85 |
|  | 153 | 1.88 | 0.33 | 10.60 |
|  | 154 | 1.84 | 0.33 | 10.33 |
|  | 155 | 1.79 | 0.32 | 10.05 |
|  | 156 | 1.73 | 0.31 | 9.76 |
|  | 157 | 1.68 | 0.30 | 9.47 |
|  | 158 | 1.62 | 0.29 | 9.18 |
|  | 159 | 1.57 | 0.28 | 8.89 |
|  | 160 | 1.51 | 0.26 | 8.61 |

Hazard ratios (HRs) and 95% confidence intervals (CIs) were estimated using Cox proportional hazard models that incorporated restricted cubic spline terms (df = 3) for systolic blood pressure (SBP). Analyses were stratified according to the presence or absence of asymptomatic brain lesions (ABL) and adjusted for age, sex, HbA1c level, LDL cholesterol level, and antihypertensive medication use. The reference SBP was set at 100 mm Hg.
