# Supplementary material for: Systolic blood pressure and future stroke risk by asymptomatic brain lesions in a community MRI cohort: a retrospective study
Source: Hypertens Res. 2026 Apr 22;49(6):1866–77. doi: 10.1038/s41440-026-02639-z (PMC13236583; doi:10.1038/s41440-026-02639-z)
Supplement: Supplementary file 4 — Supplementary Figure S1 [file 41440_2026_2639_MOESM4_ESM.docx]

**Supplementary Figure S1. Schoenfeld residual plots for testing the proportional hazards assumption in the Cox regression model**


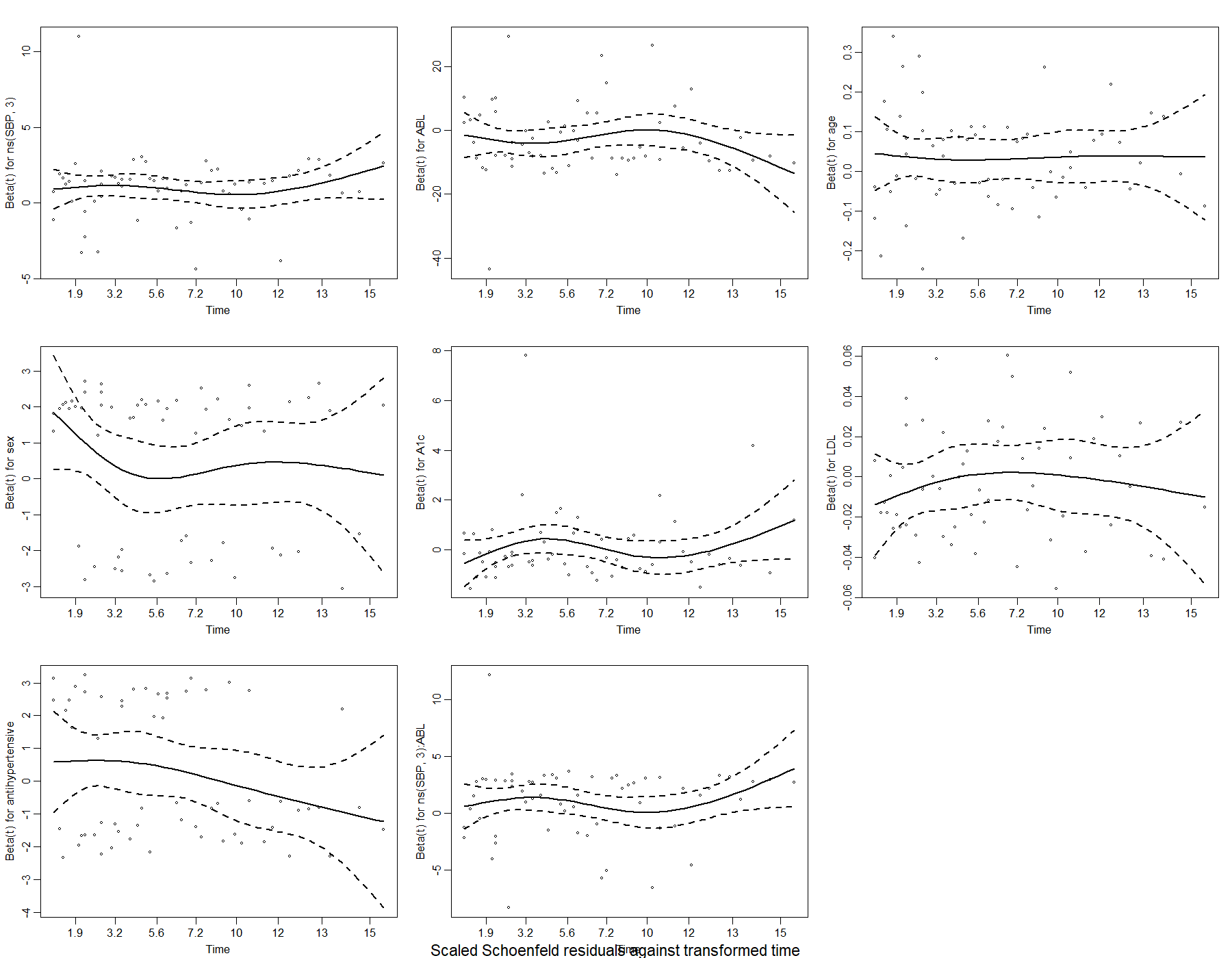


Proportional hazard diagnostics based on scaled Schoenfeld residuals for the main Cox model. Each panel shows the residuals plotted against the transformed time for one covariate together with a smoothed trend (solid line) and its 95% confidence band (dashed lines). No clear time-dependent patterns were observed, indicating that the proportional hazards assumption was reasonably satisfied by this model.
