# Supplementary material for: Systolic blood pressure and future stroke risk by asymptomatic brain lesions in a community MRI cohort: a retrospective study
Source: Hypertens Res. 2026 Apr 22;49(6):1866–77. doi: 10.1038/s41440-026-02639-z (PMC13236583; doi:10.1038/s41440-026-02639-z)
Supplement: Supplementary file 5 — Supplementary Figure S2 [file 41440_2026_2639_MOESM5_ESM.docx]

**Supplementary Figure S2. Five-year stroke risk curves for individual lesion subtypes comprising asymptomatic brain lesions (ABL).**


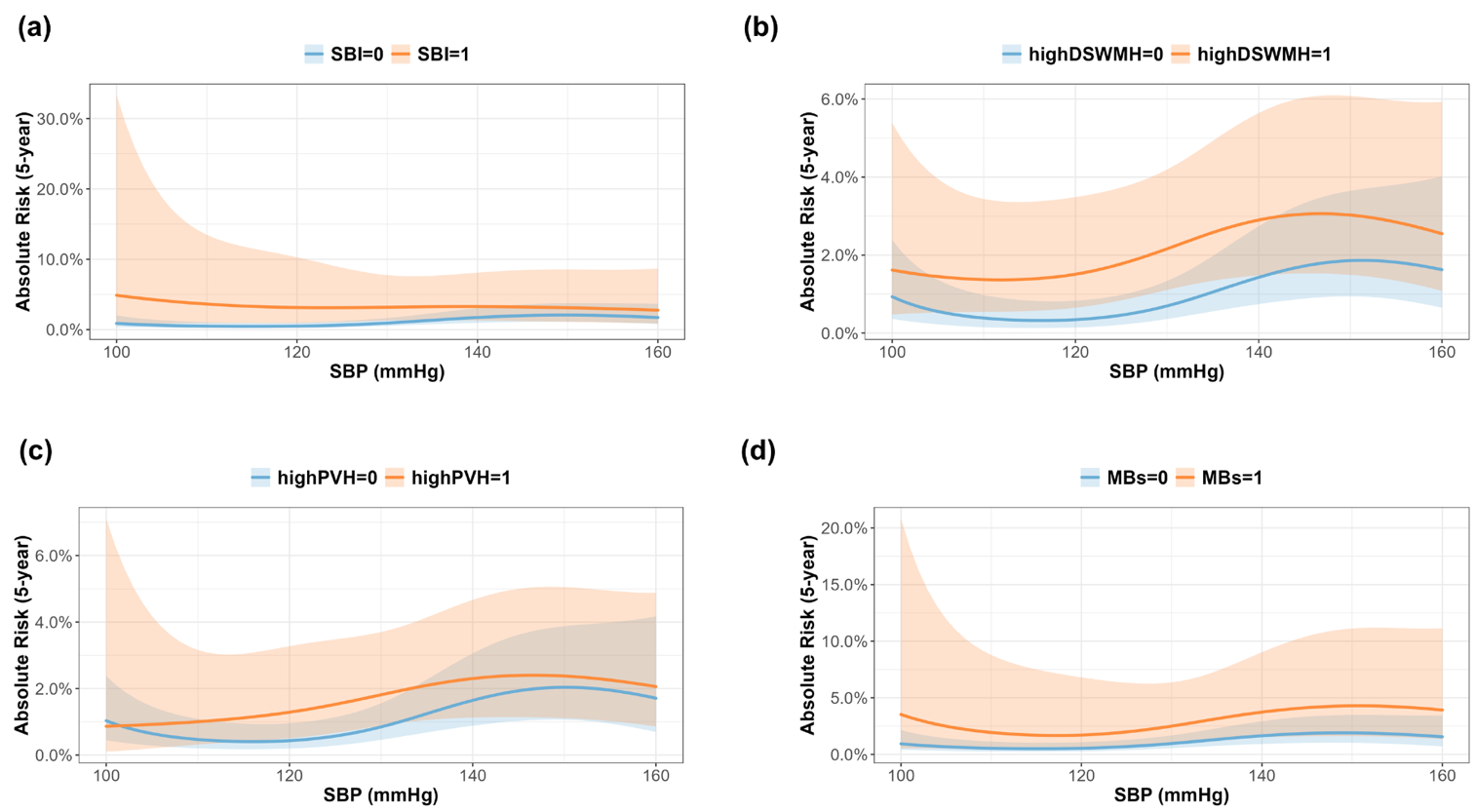


(a) Silent brain infarcts (SBI), (b) deep/subcortical white matter hyperintensity (DSWMH ≥ 2), (c) periventricular hyperintensity (PVH ≥ 2), and (d) cerebral microbleeds (CMB). Red lines represent lesion-positive participants, and blue lines represent lesion-negative participants.

Although the shapes of the spline curves differed among the lesion types, all consistently demonstrated a higher five-year stroke risk in the lesion-positive group than in the lesion-negative group.
