# Supplementary material for: Systolic blood pressure and future stroke risk by asymptomatic brain lesions in a community MRI cohort: a retrospective study
Source: Hypertens Res. 2026 Apr 22;49(6):1866–77. doi: 10.1038/s41440-026-02639-z (PMC13236583; doi:10.1038/s41440-026-02639-z)
Supplement: Supplementary file 6 — Supplementary Figure S3 [file 41440_2026_2639_MOESM6_ESM.docx]

**Supplementary Figure S3.** Restricted cubic spline curves of systolic blood pressure and stroke risk after exclusion of participants with intracranial arterial stenosis on magnetic resonance angiography.


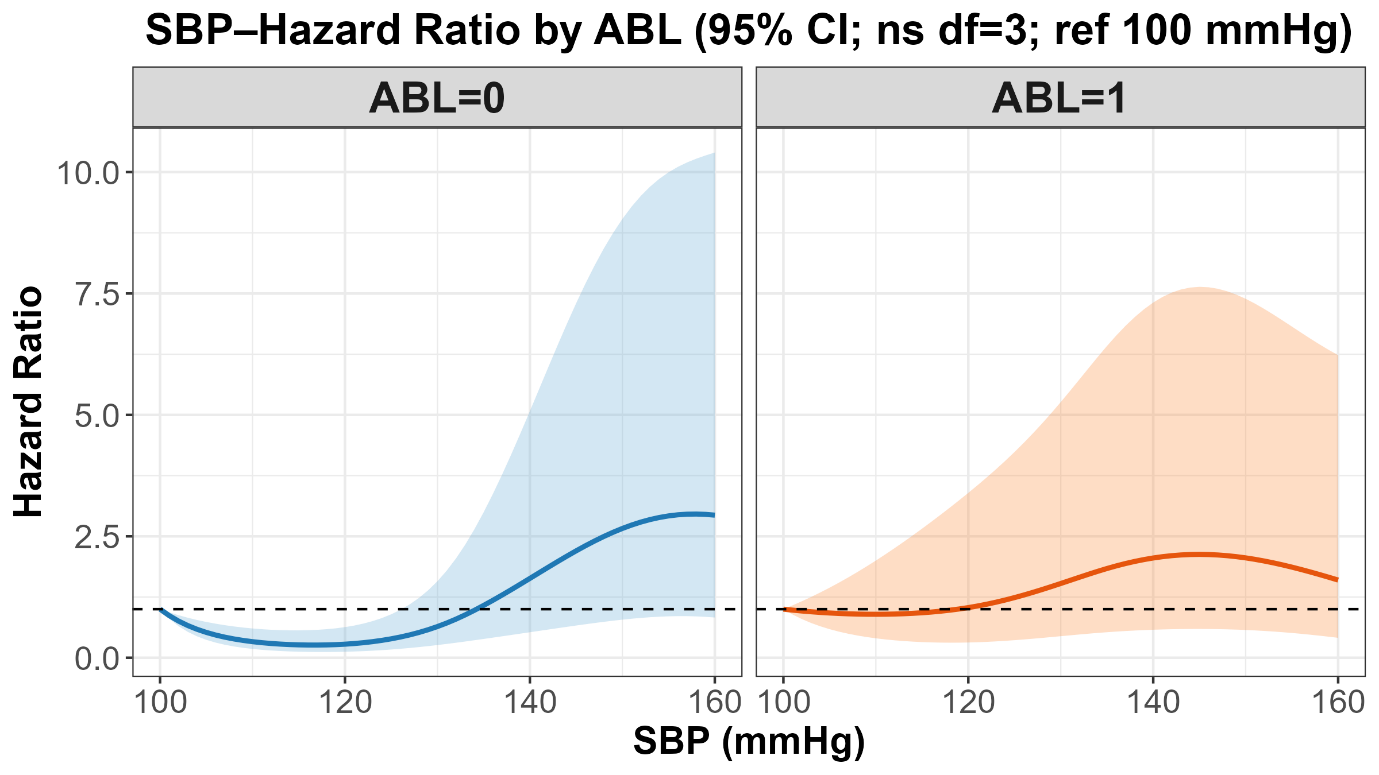


Restricted cubic spline curves (95% confidence intervals) depicting the association between systolic blood pressure and incident stroke according to asymptomatic brain lesion (ABL) status, after excluding participants with intracranial arterial stenosis detected on baseline magnetic resonance angiography.
