# Supplementary material for: Systolic blood pressure and future stroke risk by asymptomatic brain lesions in a community MRI cohort: a retrospective study
Source: Hypertens Res. 2026 Apr 22;49(6):1866–77. doi: 10.1038/s41440-026-02639-z (PMC13236583; doi:10.1038/s41440-026-02639-z)
Supplement: Supplementary file 7 — Supplementary Figure S4 [file 41440_2026_2639_MOESM7_ESM.docx]

**Supplementary Figure S4.** Restricted cubic spline curves of systolic blood pressure and stroke risk after exclusion of participants who initiated antihypertensive therapy within one year after baseline screening.


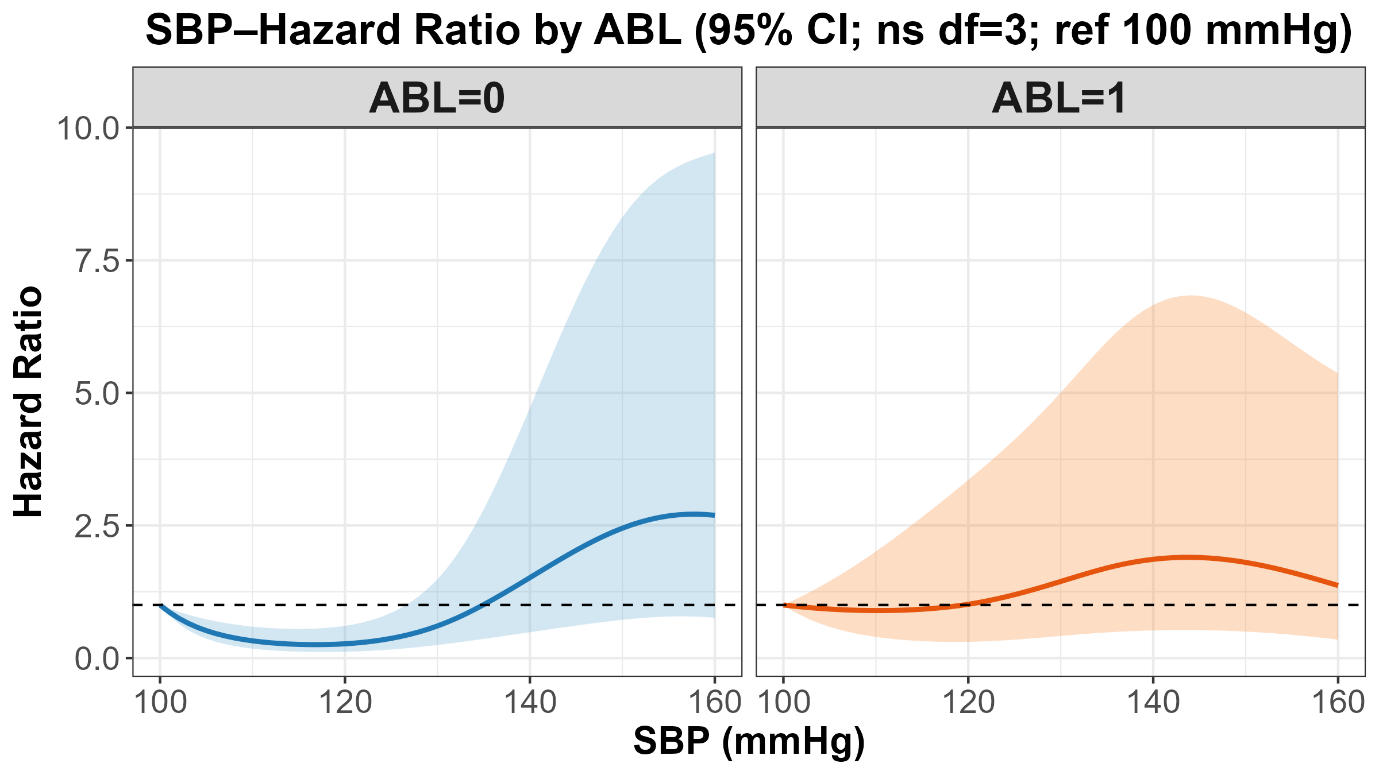


Restricted cubic spline curves (95% confidence intervals) showing the association between systolic blood pressure and incident stroke according to asymptomatic brain lesion (ABL) status, after excluding participants who initiated antihypertensive therapy within one year after baseline screening.
