# Supplementary material for: Systolic blood pressure and future stroke risk by asymptomatic brain lesions in a community MRI cohort: a retrospective study
Source: Hypertens Res. 2026 Apr 22;49(6):1866–77. doi: 10.1038/s41440-026-02639-z (PMC13236583; doi:10.1038/s41440-026-02639-z)
Supplement: Supplementary file 8 — Supplementary Figure S5 [file 41440_2026_2639_MOESM8_ESM.docx]

**Supplementary Figure S5.** Restricted cubic spline curves of systolic blood pressure and stroke risk with additional adjustment for DRS


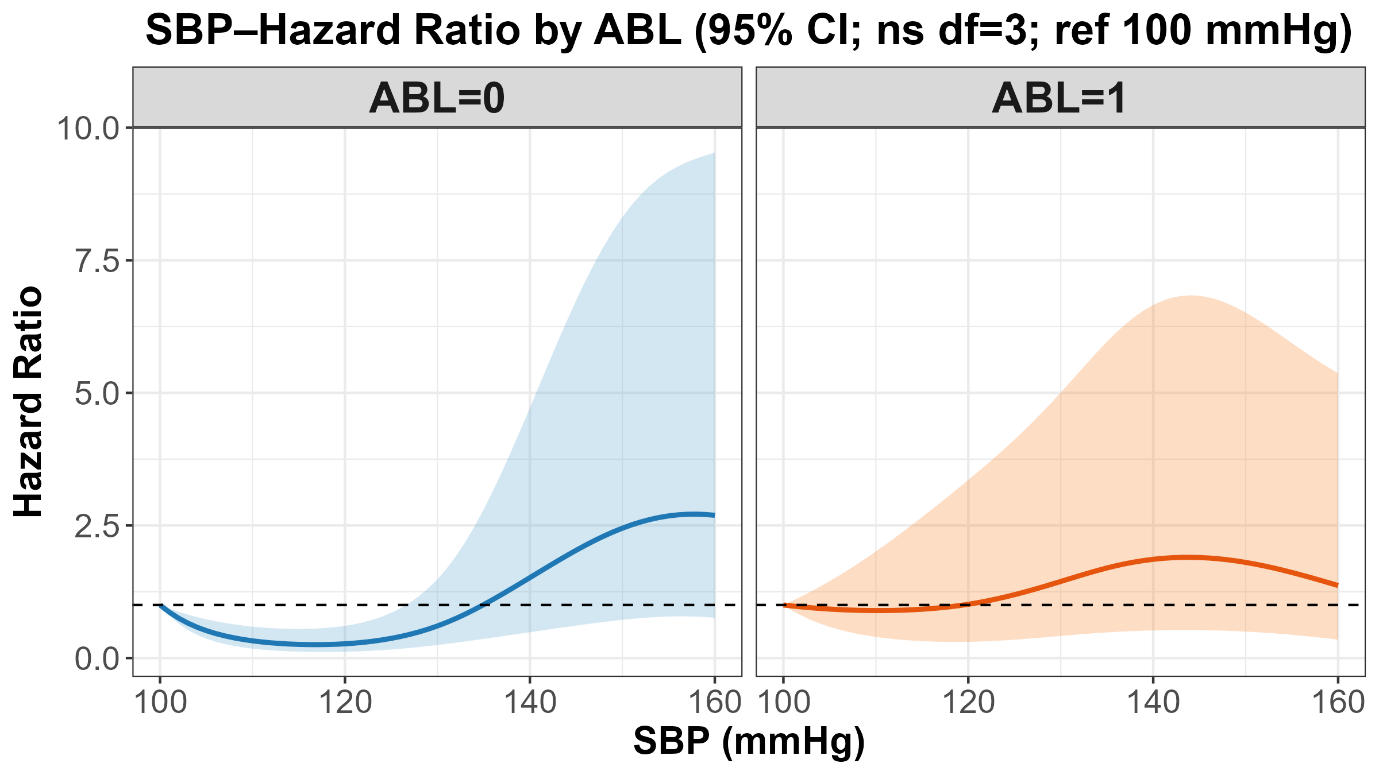


Restricted cubic spline curves (95% confidence intervals) illustrating the association between systolic blood pressure and incident stroke stratified by asymptomatic brain lesion (ABL) status, with additional adjustment for DRS.
